# In-Person and Teleconsultation Services at a National Hospital in Peru: Time Series Analysis of General and Psychiatric Care Amid the COVID-19 Pandemic

**DOI:** 10.2196/53980

**Published:** 2024-07-08

**Authors:** David Villarreal-Zegarra, Jackeline García-Serna, Piero Segovia-Bacilio, Nikol Mayo-Puchoc, Alba Navarro-Flores, Jeff Huarcaya-Victoria

**Affiliations:** 1 Escuela de Medicina Humana, Universidad César Vallejo Trujillo Peru; 2 Instituto Peruano de Orientación Psicológica Lima Peru; 3 International Max Planck Research School for Translational Psychiatry (IMPRS-TP) Munich Germany; 4 Escuela Profesional de Medicina Humana, Universidad Privada San Juan Bautista Ica Peru; 5 Unidad de Investigación de Psiquiatría, Departamento de Psiquiatría, Hospital Nacional Guillermo Almenara Irigoyen Lima Peru

**Keywords:** health care utilization, mental health use, COVID-19, mental health, health care, psychiatric care, teleconsultation, hospital, Peru, chronic, patient, patients, telemonitoring

## Abstract

**Background:**

The COVID-19 pandemic led to a global reduction in health care accessibility for both infected and noninfected patients, posing a particular burden on those with chronic conditions, including mental health issues. Peru experienced significant devastation from the pandemic, resulting in a collapsed health care system and leading to the world’s highest per capita mortality rate as a result of COVID-19. Understanding the trends in health care utilization, particularly in mental health care, is crucial for informing pandemic response efforts and guiding future recovery strategies.

**Objective:**

This study aims to analyze the trends of outpatient medical and psychiatric consultations during the COVID-19 pandemic in a national hospital in Peru.

**Methods:**

This observational study was conducted at a national hospital in Lima, Peru. We analyzed data on user care across all services, including psychiatric services, from May 2019 to December 2022. The data were calculated for users served per month, including the number of users seen monthly in mental health services. Sociodemographic variables such as sex (female or male), age (≥0 years), type of medical appointment (regular or additional), and modality of care (in-person or teleconsultations) were taken into account. An interrupted time series regression model was conducted to assess the number of outpatient medical and psychiatric consultations. Subgroup analyses were performed based on service modality, including overall consultations, telemonitoring/teleconsultations only, or face-to-face only, for all service users and for mental health service users.

**Results:**

A total of 1,515,439 participants were included, with females comprising 275,444/484,994 (56.80%) of the samples. Only 345,605/1,515,439 (22.81%) visits involved telemedicine. The total monthly outpatient visits were significantly reduced compared with the expected projection (*P*<.001) at the beginning of the pandemic, followed by a later monthly increment of 298.7 users. Face-to-face interventions experienced a significant reduction at the beginning of the pandemic (*P*<.001), gradually recovering in the following months. By contrast, telemedicine use initially increased but subsequently declined toward the end of the pandemic. A similar trend was observed in mental health units.

**Conclusions:**

During the pandemic years, health care utilization in both general and psychiatric services experienced a significant decrease, particularly at the beginning of the pandemic (March 2020). However, no significant trends were observed in either case throughout the pandemic period. Telemedicine consultations witnessed a significant increase overall during this period, particularly among mental health users.

## Introduction

During the COVID-19 pandemic, many medical consultations unrelated to the virus were significantly reduced to focus on containing the spread of the infection [1]. Health care systems faced the significant challenge of adapting to the increasing demand for treating patients with COVID-19 while continuing to provide care for those with other conditions, despite having limited resources available [2]. Outpatient clinics, in particular, experienced a reduction in utilization during this time, primarily as a result of the decrease in scheduled medical visits and the temporary suspension of some services in many health centers [3]. This situation significantly impacted patients’ health, especially those with chronic conditions requiring continuous follow-up sessions, and led to substantial economic losses [4].

In low- and middle-income countries, where access to medical care was already limited, the pandemic posed a nearly insurmountable challenge [5,6]. The prevalence of other public health issues, such as malnutrition, malaria, tuberculosis, and HIV/AIDS, rose to unprecedented levels [7,8]. The case of Peru exemplifies unmet health care needs during the pandemic in the context of a severely unprepared, yet upper-middle-income country. With hospitals suffering from poor infrastructure, limited medical supplies, and outdated technology for decades [9], Peru’s health care system collapsed during the pandemic. This led to the highest mortality rate per capita, low immunization rates, and a high frequency of infections among health care providers [10,11]. As a consequence of this situation, the outcomes for patients with chronic and noncommunicable disorders worsened [12].

An important subset of chronic patients included those with psychiatric disorders. For them, various aspects of the pandemic—such as fear of contagion, new diagnoses, and the loss of loved ones—combined with the inadequate health care response led to an increase in the incidence and severity of cases [13]. In Peru, mental health and substance abuse disorders are among the most burdensome conditions [14]. Following the COVID-19 pandemic, there has been an increase in the prevalence of moderate depressive symptoms and in the proportion of cases being treated for mild depressive symptoms [15-17].

An alternative to managing the high demand of patients was provided by telemedicine and other digital technologies, which improved accessibility and the quality of care [18,19]. However, a significant problem remains: many patients lack access to the necessary technology for telemedicine and other forms of virtual medical care utilized during the pandemic.

Hence, it is crucial to examine how the utilization of outpatient medical consultations has been affected by the COVID-19 pandemic in these countries. This exploration can help identify effective solutions to tackle present and future challenges in health care delivery. In this regard, this study will concentrate on analyzing the utilization of outpatient medical and psychiatric consultations during the COVID-19 pandemic at a social security hospital in Peru. It will use a time series analysis methodology to scrutinize trends and patterns of service utilization from 2019 to 2022.

## Methods

### Study Design

Our study adopted an observational design and utilized data from the Hospital Nacional Guillermo Almenara Irigoyen (HNGAI) in Lima, Peru.

### Setting

The study was conducted at the HNGAI, a tertiary referral center and highly complex health care facility situated in Lima, Peru. The HNGAI occupies a prominent position within Peru’s health care system, being one of the largest hospitals under the social security system in terms of bed capacity, boasting a total of 960 hospital beds. It serves as a comprehensive medical institution catering to a broad range of medical specialties, including psychiatry.

The hospital’s significance and scope of services are evident from its designation as a Specialized Health Institute III-2, the highest level bestowed by the Ministry of Health of Peru on hospital establishments. With a catchment population of 1,547,840 individuals covered by social insurance, the HNGAI plays a vital role in providing health care services to a substantial portion of the population.

As a tertiary referral center, the HNGAI assumes the responsibility for managing a diverse range of medical conditions, from routine ailments to highly complex and specialized cases. Its comprehensive capabilities and expertise make it a preferred choice for patients seeking specialized medical care.

Data on user care across all services provided by the HNGAI, including psychiatric services such as adult psychiatry, child and adolescent psychiatry, addictive behaviors, and day hospital services provided by the psychiatry department, were utilized. The data were extracted from the “ExplotaDatos system” generated by the Social Health Insurance EsSalud, covering the period from May 1, 2019, to December 31, 2022.

### Participants

The number of consultations considered for the study included all hospital services, also encompassing psychiatric services. Among these consultations, women accounted for 275,444/484,964 (56.80%) and 584,618/1,030,445 (56.73%) of the sample before and during the pandemic, respectively. The age range of participants in the study ranged from 12 to over 65 years. The number of participants with their own health insurance was 341,299/484,964 (70.38%) and 749,626/1,030,445 (72.75%) before and during the pandemic, respectively. Additionally, 391,604/484,964 (80.75%) and 911,384/1,030,445 (88.45%) participants accessed care through a regular medical appointment, while the remaining participants accessed care through an additional one. Mental health care services constituted 26,916/484,964 (5.55%) and 68,385/1,030,445 (6.64%) of the total consultations before and after the pandemic, respectively. Finally, telemonitoring and teleconsultation care accounted for 30/484,964 (0.01%) and 345,575/1,030,445 (33.54%) of the total consultations before and during the pandemic, respectively. Variables considered are listed in Textbox 1.

**Variables considered in this study.**

- 1. Users Served Per Month

Defined as the number of users served in all services during a month by means of in-person consultations as well as telemonitoring or teleconsultation.

- 2. Number of Users Seen Monthly in Mental Health Services

Defined as the number of users attended to in the mental health services, including adult psychiatry, child and adolescent psychiatry, addictive behaviors, and day hospital, through in-person consultations as well as telemonitoring or teleconsultation.

- 3. Sociodemographic Covariates

The sociodemographic variables were sex (female or male), age (≥0 years), type of medical appointment (normal or additional), and modality of care (telemonitoring or teleconsultations).

### Statistical Analysis

Descriptive analyses were conducted on users seen from May 2019 to December 2022, with monthly data collection. Sociodemographic variables were analyzed for all medical consultations, including those carried out by psychiatric services. We used interrupted time series regression models, a quasi-experimental approach, to assess the number of outpatient medical and psychiatric consultations following the onset of the COVID-19 pandemic. This involved analyzing trends and patterns of service utilization from May 2019 to December 2022. The analysis did not encompass care provided by the psychology service. The estimated impact of the pandemic on total outpatient medical and psychiatric consultations was assessed in terms of changes in level (intercept) and changes in the slope of prevalence across the time series before, during, and after the pandemic. Subgroup analyses were conducted according to service modality, that is, overall, telemonitoring/teleconsultations only, or face-to-face only, for all service users and psychiatric service users.

### Ethics Approval

We did not access any individual personal data, nor did we have contact with participants, as the data were secondary and anonymous. The data were accessed upon request, and we did not collect primary data, thus eliminating any ethical risk. Our study was approved by the Institutional Research Ethics Committee of the HNGAI (approval number 245 CIEI-IOIyD-GRPA-ESSALUD-2023).

## Results

### Participants

A total of 1,515,439 participants were attended to as outpatients between May 2019 and December 2022. During this period, the majority of participants were females (860,062/1,515,409, 56.75%), working-age adults (777,236/1,515,409, 51.29%), health insurance holders (1,090,925/1,515,409, 71.99%), and had scheduled regular medical appointments (1,302,988/1,515,409, 85.98%). Only 345,605/1,515,409 (22.81%) outpatient visits involved telemonitoring or teleconsultation. Table 1 presents the characteristics of the participants before and during the pandemic for all users and users in mental health services.

**Table 1:** Sociodemographic characteristics of the participants in all services.

Sociodemographic characteristics			All users served (n=1,515,439)					Psychiatric services (n=95,301)		
			Before the pandemic (n=484,964), n (%)		During the pandemic (n=1,030,445), n (%)^a^		Before the pandemic (n=26,916), n (%)			During the pandemic (n=68,385), n (%)^a^
**Sex**										
	Female	275,444 (56.80)		584,618 (56.73)		12,747 (47.36)			34,534 (50.50)	
	Male	209,550 (43.21)		445,827 (43.27)		14,169 (52.64)			33,851 (49.50)	
**Age categories (years)**										
	0-12	53,984 (11.13)		94,521 (9.17)		4756 (17.67)			9424 (13.78)	
	13-17	14,500 (2.99)		30,970 (3.01)		2911 (10.82)			8509 (12.44)	
	18-64	234,214 (48.30)		543,022 (52.70)		13,741 (51.05)			37,067 (54.20)	
	65 or older	182,296 (37.59)		361,932 (35.12)		5508 (20.46)			13,385 (19.57)	
**Family relationship to the insured**										
	Others	143,695 (29.63)		280,819 (27.25)		12,929 (48.03)			33,838 (49.48)	
	Holder	341,299 (70.38)		749,626 (72.75)		13,987 (51.97)			34,547 (50.52)	
**Type of medical appointment**										
	Normal	391,604 (80.75)		911,384 (88.45)		20,180 (74.97)			60,565 (88.56)	
	Additional	93,390 (19.26)		119,061 (11.55)		6736 (25.03)			7820 (11.44)	
**Telemonitoring/teleconsultations**										
	No	484,964 (100)		684,870 (66.46)		26,916 (100)			39,300 (57.47)	
	Yes	30 (0.01)		345,575 (33.54)		0 (0)			29,085 (42.53)	

^a^The lockdown started on March 16, 2020.

The most common mental health problem diagnoses in users of psychiatric services were schizophrenia, schizotypal, and delusional disorders (F20-F29); affective disorders (F30-F39); and anxiety, stress-related, and somatoform disorders (F40-F48). An extended list of the mental health diagnoses is provided in Table 2.

**Table 2:** Psychiatric diagnoses in psychiatric services (N=95,301).

ICD-10^a^ diagnostic code: diagnosis	Before the pandemic (n=26,916), n (%)	During the pandemic (n=68,385), n (%)^b^
F00-F09: Organic mental disorders including symptomatic disorders	2548 (9.47)	5622 (8.22)
F10-F19: Mental and behavioral disorders due to psychoactive substance use	2051 (7.62)	4635 (6.78)
F20-F29: Schizophrenia, schizotypal, and delusional disorders	5262 (19.55)	14,553 (21.28)
F30-F39: Mood (affective) disorders	4797 (17.82)	13,132 (19.20)
F40-F48: Neurotic, stress-related, and somatoform disorders	4297 (15.96)	11,017 (16.11)
F50-F59: Behavioral syndromes associated with physiological disturbances and physical factors	236 (0.88)	812 (1.19)
F60-F69: Adult personality and behavioral disorders	656 (2.44)	1637 (2.39)
F70-F79: Mental retardation	504 (1.87)	1197 (1.75)
F80-F89: Developmental psychological disorders	2306 (8.57)	5471 (8.00)
F90-F98: Behavioral and emotional disorders often occurring in childhood and adolescence	3485 (12.95)	5639 (8.25)
F99-F99: Mental disorder not specified	0 (0)	0 (0)
Other diagnoses	774 (2.88)	4670 (6.83)

^a^ICD-10: 10th revision of the International Statistical Classification of Diseases and Related Health Problems.

^b^The lockdown started on March 16, 2020.

### Number of Users in All Services

**Figure 1:**
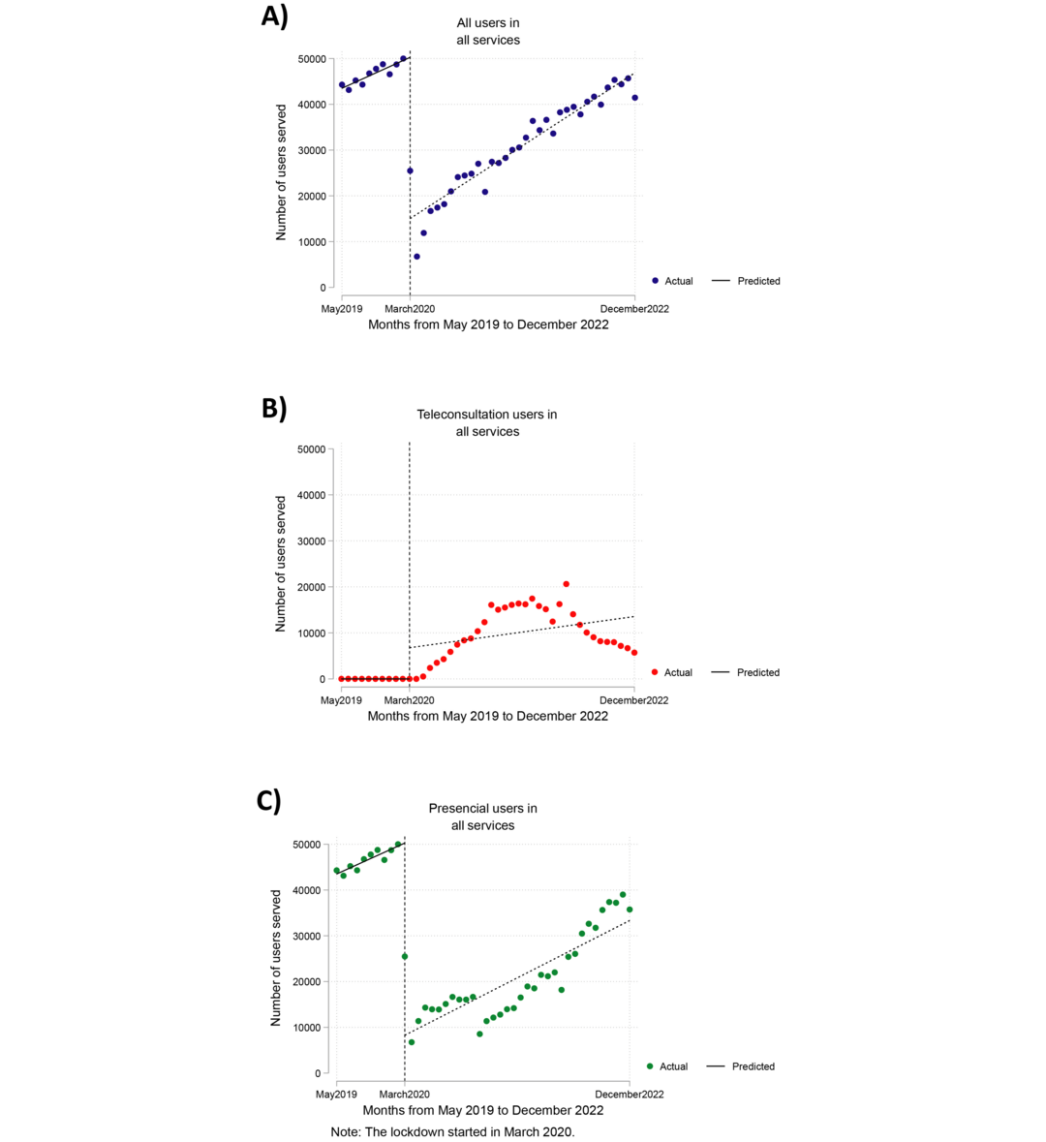
Interrupted time series analysis for the number of users served per month in all services between May 2019 and December 2022. (A) Interrupted time series analysis for users in all services. (B) Interrupted time series analysis for teleconsultation users in all services. (C) Interrupted time series analysis for face-to-face users in all services.

Figure 1A illustrates that the total number of users seen per month in outpatient care across all services experienced a significant decrease at the onset of the pandemic (March 2020), with a reduction of –35,226.4 users seen compared with what was expected for that month (95% CI –38,558.3 to –31,894.6; *P*<.001). However, an upward trend was observed during the pandemic, with a monthly increase of 289.7 users across all services (95% CI 67.2-512.2; *P*=.01). By contrast, the number of users served at the beginning of the pandemic remained the same as that measured in the month before the start of the pandemic (February 2020).

The number of users served by telemonitoring and teleconsultation was nearly 0 before the pandemic (Figure 1B). Subsequently, there was an increase in the middle of the pandemic followed by a decrease in the last months of the evaluation period. However, no significant trend was found in the number of users visited per month by telemonitoring and teleconsultation alone (*P*=.13).

The number of users served by face-to-face care per month experienced a significant reduction at the beginning of the pandemic (Figure 1C), with a decrease of –42,001.9 users served compared with what was expected for that month (95% CI –46,934.2 to –37,069.6; *P*<.001). However, no significant trend was observed in the number of users seen per month for face-to-face care alone (*P*=.58). Table 3 presents the coefficients of the time series analysis.

**Table 3:** Interrupted time series regression analysis for the number of users served.

User service				Coefficients^a^		*P* value^a^		95% CI
**All users served**								
	**Overall**							
		Preintervention slope^b^	675.5		<.001		531.5 to 819.5	
		Change in intercept^c^	–*35,226.4*		*<.001*		–38,558.3 to –31,894.6	
		Change in slope (interaction)^d^	*289.7*		*.01*		67.2 to 512.2	
		Intercept^e^	43,513.7		<.001		42,827.2 to 44,200.2	
		Postintervention linear trend^f^	965.2		<.001		812.0 to 1118.5	
	**Only telemonitoring/teleconsultations**							
		Preintervention slope	–0.8		.03		–1.6 to –0.1	
		Change in intercept	*6775.5*		*.009*		1777.9 to 11,773.0	
		Change in slope (interaction)	206.3		.13		–64.8 to 477.5	
		Intercept	6.7		.01		1.5 to 11.9	
		Postintervention linear trend	205.5		.13		–65.7 to 476.7	
	**Only in-person**							
		Preintervention slope	676.4		<.001		532.1 to 820.6	
		Change in intercept	–*42,001.9*		*<.001*		–46,934.2 to –37,069.6	
		Change in slope (interaction)	83.4		.585		–222.7 to 389.4	
		Intercept	43,507.0		<.001		42,818.3 to 44,195.8	
		Postintervention linear trend	759.7		<.001		506.5 to 1013.0	
**Psychiatric services**								
	**Overall**							
		Preintervention slope	23.2		.17		–10.5 to 56.8	
		Change in intercept	–*1550.3*		*<.001*		–1818.2 to –1282.3	
		Change in slope (interaction)	31.2		.09		–5.3 to 67.6	
		Intercept	2471.0		<.001		2326.6 to 2615.5	
		Postintervention linear trend	54.4		<.001		42.8 to 65.9	
	**Only telemonitoring/teleconsultations**							
		Preintervention slope	0.0		—^g^		—	
		Change in intercept	204.9		—		—	
		Change in slope (interaction)	39.4		—		—	
		Intercept	0.0		—		—	
		Postintervention linear trend	39.4		—		—	
	**Only in-person**							
		Preintervention slope	23.2		.17		–10.5 to 56.8	
		Change in intercept	–*1755.2*		*<.001*		–2139.2 to –1371.2	
		Change in slope (interaction)	–8.2		.67		–46.9 to 30.5	
		Intercept	2471.0		<.001		2326.6 to 2615.5	
		Postintervention linear trend	14.9		.07		–1.4 to 31.3	

^a^Values in italics are significant (*P*<.05).

^b^Preintervention slope corresponds to the previous trend of the number of users served.

^c^Change in intercept refers to the change in the number of users served at the beginning of the COVID-19 lockdown.

^d^Change in slope (interaction) refers to the change in the trend of the number of users served over time after March 2020.

^e^Intercept represents the number of users served at the beginning of the study period.

^f^Postintervention linear trend represents the trend in the number of users served after the onset of the pandemic. Autocorrelation at lag(1) was considered.

^g^The model did not converge so the analysis as such could not be performed.

### Number of Users in Psychiatric Services

**Figure 2:**
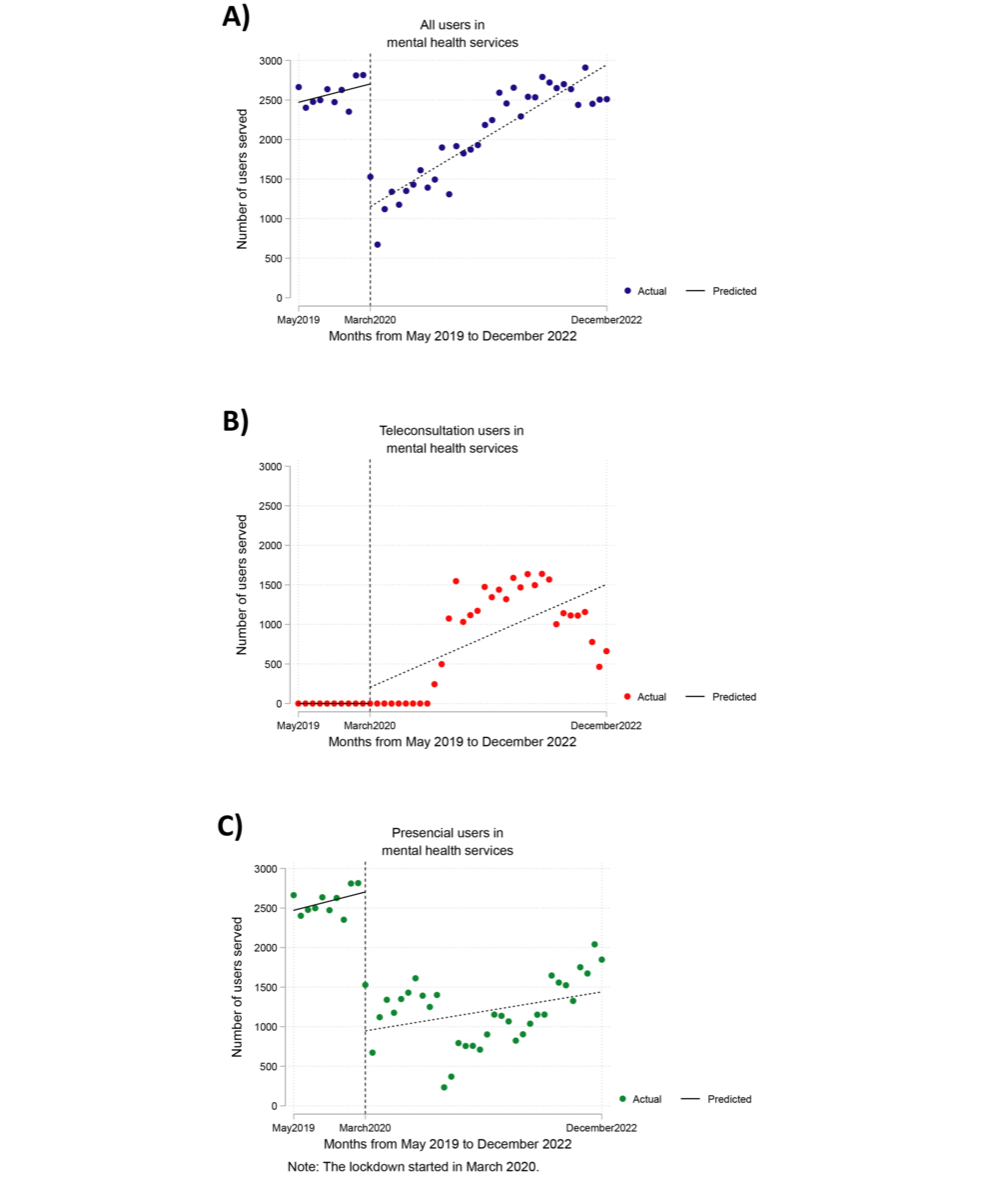
Interrupted time series analysis for the number of users served per month in all psychiatric services between May 2019 and December 2022. (A) Interrupted time series analysis for users in mental health services. (B) Interrupted time series analysis for teleconsultation users in mental health services. (C) Interrupted time series analysis for face-to-face users in mental health services.

Figure 2A illustrates that the number of outpatients seen per month in the 4 psychiatric services experienced a significant reduction at the onset of the pandemic (March 2020). There were –1550.3 fewer users seen than expected for that month (95% CI –1818.2 to –1282.3; *P*<.001). However, although the number of users seen per month increased during the pandemic, no significant trend was found (*P*=.09).

The number of users receiving telemonitoring and teleconsultation services in the 4 psychiatric services per month is indicated in Figure 2B. However, the time series analysis did not converge, making it impossible to assess whether there were significant changes (Table 3).

There was a significant reduction in the number of face-to-face user visits per month in the 4 psychiatric services at the beginning of the pandemic (Figure 2C). The reduction amounted to –1755.2 users compared with the expected number for that month (95% CI –2139.2 to –1371.2; *P*<.001). However, no significant trend was found during the pandemic for the number of face-to-face visits per month only (*P*=.67).

## Discussion

### Principal Findings

During the initial months of the pandemic, there was a decrease in the number of users accessing health services, which gradually increased over time. Before the pandemic, medical care through telemonitoring and teleconsultation was almost nonexistent, but it increased during the pandemic before decreasing again in the last months of the evaluated period. By contrast, there were no significant differences in the number of users attending face-to-face, telemonitoring, and teleconsultation modalities during the evaluation months. Regarding patients seen in psychiatric services, we observed a significant decrease in the number of outpatients at the beginning of the pandemic, followed by a progressive increase over time. Face-to-face visits per month were significantly reduced in all 4 mental health services.

### Comparison With Other Studies

At the beginning of the pandemic, we observed an overall reduction in health care utilization. A systematic review conducted until August 2020, encompassing 20 countries, found that health care utilization was reduced in one-third of them. Eighty-one primary studies reported 143 estimates and concluded a mean reduction of 28% in admissions and 31% in new diagnoses [1]. In Peru, mandatory confinement was established in March 2020, lasting approximately 3 months [20]. During this period, the population was not allowed to leave their homes except for emergencies.

Amid the pandemic, we observed a gradual but slow increase in care utilization. A study using a time series design to assess the effect of the pandemic on 31 health services across 10 countries (low income=2, lower middle income=3, upper middle income=3, and high income=2) found that total outpatient visits decreased between 9% and 40% and remained below expectations by the end of 2020 [21]. A scoping review analyzing changes in medical care access found that among 38 studies from Europe, Asia, and Africa, 33 reported a statistically significant reduction in service use. Similarly, the studies reported possible barriers to health care access, including limited supplies and personnel to care for patients with non–COVID-19 having other medical issues, as well as increased waiting times. A potential explanation for this slow increment in Peru, as well as in other Latin American countries, could be provided by a survey reporting that around 66% of Peruvian participants attempted self-medication during the pandemic [22]. Additionally, part of the population faced barriers such as fear of contagion and the stigma of being diagnosed with COVID-19, as well as financial difficulties [23]. Although the number of cases was not increasing during and after the first phase of restriction, the maintenance of low rates of utilization was suggested to be a result of factors such as fear of contagion, sanctions related to outside mobilization, reduced access to medical centers, and prioritization of COVID-19 cases, among others [24]. We observed that by the end of the pandemic, overall health care utilization rates had risen to levels comparable to those observed 1 year before the pandemic’s onset. However, a notable addition was the emergence of a new category of consultations conducted via telemedicine. Similarly, a study in the United Kingdom found that after the release of social restriction measures, the frequency of health care utilization returned to levels that were not significantly different from prepandemic utilization [25].

Regarding psychiatric services utilization, we observed a reduction at the beginning of the pandemic that progressively improved toward the end of this period. Amidst the pandemic, Peru was submerged in chaos because of various factors, including a debilitated health care system, political mismanagement, and poor adherence to social restrictions [11]. This scenario significantly impacted the mental health of the population, as evidenced by high levels of perceived stress in the general Peruvian population [26]. A time series analysis of depression diagnoses in Peru showed that the number of new patients increased by 0.17% per month after the beginning of the pandemic [15].

In the United States, during the initial months of the pandemic, there was a 50% reduction in face-to-face mental health encounters. However, the utilization of telehealth services played a pivotal role in the swift restoration of service delivery. This uptake of telehealth, accounting for approximately 47.9% of average monthly encounters, facilitated the timely provision of mental health care despite the limitations imposed by the pandemic [27]. In another study, it has been reported that the COVID-19 pandemic resulted in a significant decrease of more than 50% in in-person mental health care utilization rates among commercially insured adults. Rates of various mental health disorders, including anxiety disorders, bipolar disorder, and adjustment disorders, declined during the pandemic. By contrast, telehealth service utilization increased substantially, by 16-20 times. Combining both in-person and telehealth services, there was an overall increase in care observed for anxiety and adjustment disorders. These findings highlight the impact of the pandemic on mental health care delivery and the potential of telehealth in providing accessible services during times of crisis [28].

In the domain of psychiatric services, there was a discernible decline of 12% per week in visits to psychiatric emergency wards during the initial phase of social distancing measures implemented from March to May 2020 [29]. In France, an analysis of the number of medical admissions to a psychiatric ward found that, compared with 2019, admissions were reduced by 18% in 2020, with this reduction increasing to 42% during the first lockdown. Similarly, the number of patients admitted to the emergency ward was reduced by 20% and 56%, respectively [30]. A study in Italy showed that 25% of their community mental health centers reduced their access hours [31]. Conversely, a Peruvian study found that community mental health centers were able to regain service capacity and fill the service gap created by the health crisis 9 months after the COVID-19 pandemic [32].

In Peru, telemedicine and in-person health care showed a similar trend compared with other countries in the region. In many Latin American countries, efforts to establish a telemedicine system produced positive results, as they reduced the overload of hospitals, decreased waiting times, and provided access to patients living in remote areas [33]. A study conducted in an emergency department in Argentina showed that telemedicine consultations peaked during the first lockdown period of the COVID-19 pandemic in March 2020. Subsequently, telemedicine consultations reduced progressively and were replaced by face-to-face interventions, but they remained at a stable value above the prepandemic trend [34]. It is worth noting that most of the teleconsultations were conducted without technical complications. Telemedicine in our context has been supported by a legal framework since 2017, and after the pandemic outbreak, it was included as part of several regulations of the Ministry of Health and the Peruvian College of Medicine, demonstrating the interest in the establishment and refinement of these technologies in health care [35]. The transition of mental health services to telemedicine in response to high patient demand has demonstrated that synchronous digital interventions facilitate the continuity of care [36]. These interventions not only reduce geographic limitations for patients and therapists but also eliminate the long hours of travel common in Lima [36].

Additionally, these interventions reduce stigma because they occur in a more private setting, can be delivered by trained health care providers (not only physicians), and could reduce costs [37]. A survey of psychologists in the United States showed an 85% increase in the use of telemedicine after the pandemic, and they projected that at least 35% of their work would involve telemedicine after the pandemic [38]. In addition, they mentioned that telemedicine use by psychologists was positively influenced by (1) being female; (2) having training in that field and treating patients with anxiety or partners’ or women’s complaints; and (3) working in a rural area, treating patients with antisocial personality disorder, or doing rehabilitation or psychometry work. Although telemedicine offers several advantages, it is crucial to consider the values and preferences of both patients and therapists to improve utilization rates and ease the demand for in-person care.

Our study revealed a decrease in mental health services during the latter months of 2022, potentially attributable to reduced utilization of teleconsultations within the evaluated health care system following the easing of COVID-19 pandemic restrictions. This could be attributed to the inconsistent implementation of mental health teleconsultations within the Peruvian context. Professionals often lacked dedicated equipment for these consultations, and there was an overwhelming demand for care, resulting in difficulties securing teleconsultation appointments [39]. Furthermore, patients with mental health issues expressed a preference for face-to-face care over teleconsultation care [40]. These factors likely contributed to the observed decrease in mental health teleconsultations following the resolution of the health emergency.

### Public Health Implications

These results indicate that the decline in health care utilization during the pandemic underscores the fragility of the health care system. It was ill-prepared not only for managing a pandemic but also for delivering care in routine circumstances [41]. This calls for an improvement of the structure of the health care system, such as enhancing the surveillance system, training professionals for emergencies and disasters, strengthening the relationships between scientific and medical institutions, and fostering community engagement [42].

The use of telemedicine as a potential intervention to alleviate the strain on hospitals and health care centers has been a notable aspect of the pandemic response, underscoring the importance of implementing strategies for its adoption. This includes providing proper training to relevant stakeholders on these topics [43]. Future studies could explore the trends in health care utilization, particularly the significant uptake of telemedicine, which was virtually nonexistent before and during the peak of the pandemic but showed a progressive reduction (without disappearing entirely) toward the latter stages of analysis.

Furthermore, we encourage policy makers and health intelligence teams to utilize our findings as a foundation for developing health policies and regulations aimed at enhancing teleconsultation in mental health and health care overall. In Peru, the implementation of teleconsultation posed significant challenges. Many health professionals lacked the necessary equipment for conducting consultations, there was a lack of training in teleconsultation platform usage, and care centers themselves had limited access to the internet [39,44]. Moreover, telemedicine users persisted even after the pandemic, underscoring the ongoing demand for such services in resource-constrained settings such as Peru. Authors from countries sharing similar contexts could draw lessons from our positive telemedicine experience. We advocate for the implementation of these technologies within a framework that considers both barriers and facilitators [37]. Our findings can also be used to assess the postpandemic recovery of the health system.

### Limitations and Strengths

This study highlights several strengths. First, it analyzes data from a leading national reference hospital in Lima, Peru, using a time series analysis to track utilization trends. We encompassed all patients receiving care over an extensive period, spanning most of the pandemic’s duration. The sample size exceeded 1.5 million patients, and we also acquired additional data on mental health utilization, acknowledging its substantial burden in Peru. Advanced statistical methods were rigorously used in the methodology to analyze trends in health care utilization over these years. However, it is important to note that these results are confined to a national hospital in Lima and may not apply to other areas of the country or region. Further exploration of determinants affecting the variability in health care utilization, such as demographic, epidemiological, or clinical factors, is warranted. Therefore, we recommend further studies to assess whether care varied among specific age groups, genders, educational levels, or clinical variables, which may have hindered participants from accessing care.

### Conclusions

In this time series analysis conducted at a national hospital in Peru during the pandemic years (March 2020 to December 2022), data from 1.5 million patients were analyzed. We observed a significant decrease in face-to-face health care utilization at the onset of the pandemic (March 2020) in both the outpatient clinic and mental health care services, indicating a notable impact on patient attendance. No significant trends were observed in both groups over the course of the pandemic. However, there was a noticeable increase in the number of users accessing mental health services each month. Overall, there was a trend of increased monthly utilization of the aforesaid services among all users during the pandemic period. Notably, telemedicine interventions were virtually nonexistent before the pandemic, but consultations during this period increased significantly, both overall and particularly among mental health users. At the conclusion of the study period, telemedicine services in mental health continued to attract users, indicating sustained demand for these interventions that were previously unavailable before the pandemic. Further follow-up evaluations could assess the long-term feasibility of this intervention as a means to enhance mental health access in the country. The utilization notably decreased in the subsequent months, as evidenced by the decline in the number of users accessing health care services at the hospital and in the number of teleconsultation users across all services. During times of emergency, the availability of easily accessible care services and the implementation of digital health services have proven to be of utmost importance. These results serve as a demonstration of the profound impact of the national COVID-19 lockdown on health care utilization in Peru. Governmental and key decision makers are encouraged to respond promptly to health needs and to draw lessons from these experiences, thus aiding the population in facing future health emergencies effectively.

## Abbreviations

HNGAI: Hospital Nacional Guillermo Almenara Irigoyen

## Availability of Data and Materials

The data used or analyzed in this study are available from the corresponding author on reasonable request.
